# A comprehensive physical functional assessment of survivors of critical care unit stay due to COVID-19

**DOI:** 10.62675/2965-2774.20240284-en

**Published:** 2024-04-16

**Authors:** Marcia Souza Volpe, Ana Carolina Cardoso dos Santos, Sílvia Gaspar, Jade Lara de Melo, Gabriela Harada, Patrícia Rocha Alves Ferreira, Karina Ramiceli Soares da Silva, Natália Tiemi Simokomaki Souza, Carlos Toufen, Luciana Dias Chiavegato, Marcelo Britto Passos Amato, Maria Ignez Zanetti Feltrim, Carlos Roberto Ribeiro de Carvalho

**Affiliations:** 1 Universidade Federal de São Paulo Department of Human Movement Sciences Santos SP Brazil Department of Human Movement Sciences, Universidade Federal de São Paulo - Santos (SP), Brazil.; 2 Universidade de São Paulo Faculdade de Medicina Hospital das Clínicas São Paulo SP Brazil Division of Pneumology, Instituto do Coração, Hospital das Clínicas, Faculdade de Medicina, Universidade de São Paulo - São Paulo (SP), Brazil.; 3 Universidade de São Paulo Faculdade de Medicina Hospital das Clínicas São Paulo SP Brazil Department of Physiotherapy, Instituto do Coração, Hospital das Clínicas, Faculdade de Medicina, Universidade de São Paulo - São Paulo (SP), Brazil.; 4 Universidade Federal de São Paulo Discipline of Pneumology São Paulo SP Brazil Discipline of Pneumology, Universidade Federal de São Paulo - São Paulo (SP), Brazil.

**Keywords:** COVID-19, Coronavirus infections, Critical care, Recovery of function, Respiratory function tests

## Abstract

**Objective:**

To examine the physical function and respiratory muscle strength of patients - who recovered from critical COVID-19 – after intensive care unit discharge to the ward on Days one (D1) and seven (D7), and to investigate variables associated with functional impairment.

**Methods:**

This was a prospective cohort study of adult patients with COVID-19 who needed invasive mechanical ventilation, non-invasive ventilation or high-flow nasal cannula and were discharged from the intensive care unit to the ward. Participants were submitted to Medical Research Council sum-score, handgrip strength, maximal inspiratory pressure, maximal expiratory pressure, and short physical performance battery tests. Participants were grouped into two groups according to their need for invasive ventilation: the Invasive Mechanical Ventilation Group (IMV Group) and the Non-Invasive Mechanical Ventilation Group (Non-IMV Group).

**Results:**

Patients in the IMV Group (n = 31) were younger and had higher Sequential Organ Failure Assessment scores than those in the Non-IMV Group (n = 33). The short physical performance battery scores (range 0 - 12) on D1 and D7 were 6.1 ± 4.3 and 7.3 ± 3.8, respectively for the Non-Invasive Mechanical Ventilation Group, and 1.3 ± 2.5 and 2.6 ± 3.7, respectively for the IMV Group. The prevalence of intensive care unit-acquired weakness on D7 was 13% for the Non-IMV Group and 72% for the IMV Group. The maximal inspiratory pressure, maximal expiratory pressure, and handgrip strength increased on D7 in both groups, but the maximal expiratory pressure and handgrip strength were still weak. Only maximal inspiratory pressure was recovered (i.e., > 80% of the predicted value) in the Non-IMV Group. Female sex, and the need and duration of invasive mechanical were independently and negatively associated with the short physical performance battery score and handgrip strength.

**Conclusion:**

Patients who recovered from critical COVID-19 and who received invasive mechanical ventilation presented greater disability than those who were not invasively ventilated. However, they both showed marginal functional improvement during early recovery, regardless of the need for invasive mechanical ventilation. This might highlight the severity of disability caused by SARS-CoV-2.

## INTRODUCTION

Coronavirus disease 2019 (COVID-19) with acute respiratory failure has been associated with prolonged intensive care unit (ICU) stays and sustained functional impairment.^(1)^ However, the functional impairment of patients surviving severe and critical forms of COVID-19 is still poorly reported, with most data coming from the first wave of the pandemic and involving older patients.^(2–5)^ Moreover, most studies were retrospective, and there are limited studies on respiratory muscle strength in this population.^(6,7)^

Although critical and moderate cases of COVID-19 have practically ceased, it has been suggested that functional impairment among survivors in the ICU due to COVID-19 may not substantially differ from that among survivors recovering from critical illness caused by other factors.^(8)^ Consequently, understanding the respiratory and physical functioning of patients in the initial phases of recovery from severe COVID-19 could guide the development of interventions and therapies to support rehabilitation not only for patients recovering from COVID-19 but also for those recovering from critical illnesses unrelated to COVID-19.

The primary aim of this study was to examine the physical function and respiratory muscle strength of patients who recovered from critical COVID-19 after ICU discharge to the ward on days one and seven. The secondary aim was to investigate variables associated with physical impairment.

## METHODS

We conducted a prospective cohort study in two Brazilian hospitals, *Instituto do Coração* of the *Hospital das Clínicas* of the *Faculdade de Medicina* of the *Universidade de São Paulo* (USP) and *Hospital São Paulo*, *Universidade Federal de São Paulo* (UNIFESP), Brazil. This study was approved by the Ethics Committees at both hospitals (*Hospital das Clínicas* of the *Faculdade de Medicina* of the USP: n° 4.711.382 and UNIFESP: n° 4.870.812). Informed consent was obtained from all patients.

No sample size calculation was performed due to the exploratory nature of the study, and a convenience sample was used. The inclusion criteria were patients admitted to the ICU due to laboratory-confirmed COVID-19 infection, age ≥ 18 years, need for invasive mechanical ventilation (IMV), non-invasive ventilation (NIV) or high-flow nasal cannula, and ICU discharge to the ward. The exclusion criteria were a history of amputation of the hand of the dominant upper limb, previous and permanent cognitive disorders or neuromusculoskeletal deficits, inability to consent to participate in the study and/or inability to complete the proposed physical evaluations. Enrollment occurred between July 2021 and February 2022.

The study participants underwent the following assessments on the first day (D1) in the ward after ICU discharge and on the seventh day (D7), or earlier if the patient would be discharged from the hospital: short physical performance battery (SPPB), Medical Research Council sum-score (MRC-SS), ICU mobility scale, handgrip strength, maximal inspiratory pressure (MIP), and maximal expiratory pressure (MEP).

The SPPB combines three functional tests—standing balance, 3-meter gait speed, and 5-repetition sit-to-stand. SPPB scores from 0 - 3 indicate severe impairment, 4 - 6 indicate low function, 7 - 9 indicate intermediate function, and 10 - 12 indicate normal function.^(9)^ The MRC-SS ranges from 0 (total paralysis) to 60 (normal strength) and was used to evaluate global muscle strength according to a standardized protocol.^(10)^ The ICU mobility scale is an 11-point scale used to measure the highest level of functional mobility of patients, where 0 means no mobility and 10 means walking independently without a gait aid.^(11)^ Handgrip strength from the dominant hand was assessed according to a standardized protocol^(12)^ and was reported as a percentage of reference values.^(13)^ The MIP and MEP were evaluated according to the American Thoracic Society (ATS) guidelines^(14)^ and are reported as percentages of the predicted values.^(15)^

Participants were grouped according to the need for IMV into an IMV Group and a Non-IMV Group. Categorical variables are reported as counts and percentages. Continuous variables are reported as the mean and standard deviation or medians and interquartile ranges, according to the distribution. Categorical variables were compared using the chi-square test or Fisher's exact test, as appropriate. Continuous variables were compared using *t* tests, Wilcoxon-Mann–Whitney tests, or analysis of variance for repeated measures, as recommended. The analysis of variance model was built with one within factor (time: D1 *versus* D7) and one between factor (Groups IMV *versus* Non-IMV). Significant variables (p ≤ 0.05) were included in multiple regression analysis models to investigate predictors of physical function according to SPPB and handgrip strength. Missing data were not imputed. A 2-sided *p* value < 0.05 was considered to indicate statistical significance.

## RESULTS

Sixty-four patients were included in the study, 33 of whom required IMV. On D7, 11 patients (7 from the Non-IMV Group and 4 from the IMV Group) were not evaluated because of unscheduled hospital discharge. Table 1 presents the demographics and clinical characteristics of the participants in both groups. Patients in the IMV Group were younger, had higher SOFA scores, and had less hypertension than patients who did not require IMV.

**Table 1:** Demographics and clinical characteristics of participants in the Invasive Mechanical Ventilation and Non-Invasive Mechanical Ventilation Groups

		Non IMV (n = 31)	IMV (n = 33)	p value
Age (years)		61.4 ± 14.3	51.3 ± 15.5	0.009*
Weight (kg)		80.3 ± 18.3	83.1 ± 22.4	
BMI (kg/m^2^)		28.8 ± 5.0	28.9 ± 6.7	
Male (sex) n (%)		19 (61.3)	21 (63.6)	
Comorbid conditions				
	Hypertension	16 (51.6)	7 (21.2)	0.018†
	Obesity	7 (22.6)	12 (36.4)	
	Cardiovascular disease	8 (25.8)	4 (12.1)	
	Dyslipidemia	10 (32.3)	8 (24.2)	
	Cigar history	10 (32.3)	7 (21.2)	
	Kidney disease	5 (16.1)	10 (30.3)	
	COPD	3 (9.7)	2 (6.1)	
Number of comorbid conditions				
	0	2 (6.5)	6 (18.2)	
	1	12 (38.7)	13 (39.4)	
	2	6 (19.4)	8 (24.2)	
	3	8 (25.8)	3 (9.1)	
	≥ 4	3 (9.7)	3 (9.1)	
Need of Hemodialysis		2 (6.5)	12 (36.4)	0.006†
SOFA, Day 1 at ICU		2 [2 - 4]	7 [3 - 10.5]	< 0.001‡
SOFA, Day 5 at ICU		2 [2 - 5]	7 [3 - 11]	< 0.001‡
Corticosteroids use§ (days)		13 [9 - 18]	25 [16 - 30.0]	< 0.001‡
ICU stay (days)		11 [8 - 16]	29 [16 -47.5]	< 0.001‡

IMV - invasive mechanical ventilation; BMI - body mass index; COPD - chronic obstructive pulmonary disease; SOFA - Sequential Organ Failure Assessment; ICU - intensive care unit.

* t test;

† Fisher exact test or chi-square test;

‡ Mann-Whitney U test;

§ Corticosteroid use evaluation was limited to 30 days of hospital stay. Results expressed as mean ± standar deviation, n (%) or median [interquartile range]

In the Non-IMV Group, the majority (54.8%) of patients used high-flow nasal cannula (HFNC), followed by HFNC interspaced with NIV (35.5%), and a minority (9.7%) used only NIV. The median duration of HFNC therapy was 6 [4 - 8] days, while NIV lasted for 3 [1 - 8] days. In the IMV Group, 72.7% of patients required an average of 2.0 (1.0) prone positioning sessions. The median duration of neuromuscular blockers use during controlled IMV was 3.0 [1 - 7] days, and it was 0.5 [0 - 3] days during assisted IMV. Tracheostomy was performed in 42.4% of patients, and the duration of IMV was 15 [9 - 38] days.

**Figure 1:**
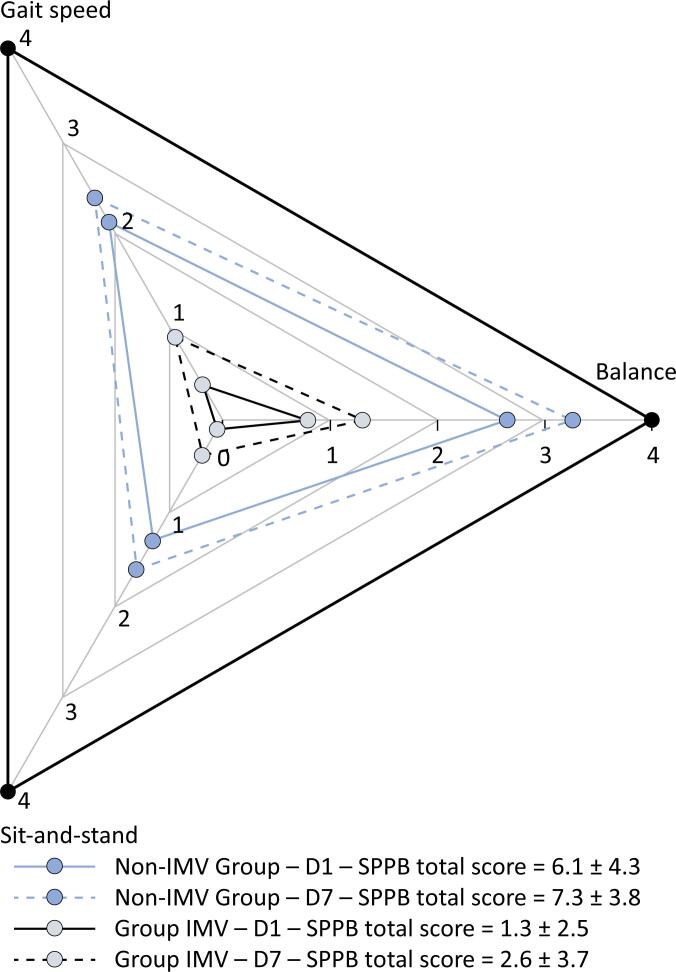
Short physical performance battery diagram showing the subscores for three tests—standing balance, gait speed, and sit-and-stand—of both groups on Days 1 and 7.

Table 2 presents the results of the physical assessments of the patients in both groups on Days 1 and 7 in the ward after ICU discharge. There was a slight improvement in the SPPB score over time in both groups (p ≤ 0.001, factor time), but the SPPB score was still poor, especially in the IMV Group (p ≤ 0.001, factor group). Figure 1 presents a diagram with the subscores for the three functional tests—standing balance, gait speed, and sit-and-stand—that compose the SPPB summary score. Clearly, the lowest scores for both groups were from the sit-and-stand test.

**Table 2:** Results of physical functioning assessments at Days 1 and 7 in the Invasive Mechanical Ventilation Group and Non Invasive Mechanical Ventilation Group

	Non IMV (n = 24)		IMV (n = 29)		p value*	p value†
	Day 1	Day 7	Day 1	Day 7		
SPPB (pts)	6.1 ± 4.3	7.3 ± 3.8	1.3 ± 2.5	2.6 ± 3.7	≤ 0.001	≤ 0.001
MRC-SS (pts)	54.7 ± 7.3	55.8 ± 6.4	42.6 ± 11.0	43.7 ± 10.6	≤ 0.001	0.209
ICU mobility (pts)	8.0 ± 2.3	9.2 ± 1.9	4.6 ± 2.3	6.1 ± 2.9	≤ 0.001	≤ 0.001
HGS, % of predicted	53.0 ± 20.5	54.0 ± 21.2	24.1 ± 17.1	30.0 ± 16.8	≤ 0.001	0.005
MIP, % of predicted	85.9 ± 39.5	99.1 ± 37.1	55.3 ± 31.1	64.5 ± 33.4	0.002	0.007
MEP, % of predicted	56.4 ± 28.7	66.3 ± 24.6	36.7 ± 20.2	42.9 ± 20.2	0.001	0.028

IMV - invasive mechanical ventilation; SPPB - short physical performance battery; MRC-SS - Medical Research Council sum score; ICU - intensive care unit; HGS - handgrip strength; MIP - maximal inspiratory pressure; MEP - maximal expiratory pressure.

* Analysis of variance for repeated measures, between factor (Groups Invasive Mechanical Ventilation *versus* Non-Invasive Mechanical Ventilation).

† Analysis of variance for repeated measures, within factor (time, Day 1 *versus* Day 7). Results expressed as mean ± standard deviation, when not otherwise indicated.

Each test score varies from 0 to 4, and the short physical performance battery summary score varies from 0 to 12. Scores between 0 and 3 denote severe physical function impairment, scores between 4 and 6 indicate low function, scores between 7 and 9 indicate intermediate function, and scores between 10 and 12 indicate normal function. The Invasive Mechanical Ventilation Group scores are presented in gray, and the Non-Invasive Mechanical Ventilation Group scores are presented in blue. The Day 1 scores are shown as continuous lines, and the Day 7 scores are shown as dashed lines. The data are presented as the mean ± standard deviation.

IMV - invasive mechanical ventilation; SPPB - short physical performance battery; D1 - Day 1; D7 - Day 7.

Neither group presented a significant increase in MRC-SS scores over time (p = 0.209). The incidence of intensive care unit-acquired weakness (MRC-SS < 48) was 13% on D7 for the Non-IMV Group, while in the IMV Group, it was 72%.

The ICU mobility scale score increased in both groups (p ≤ 0.001, factor time). In the Non-IMV Group, the mean score ranged from "walking with assistance of 1 person" to "walking independently with a gait aid", and in the IMV Group, the mean score ranged from "transferring from bed to chair" to "marching on spot at bedside" (Table 2).

Interestingly, the MIP was less affected, while the MEP and handgrip strength were well below 80% of the predictive values, especially in the IMV Group (Table 2). MIP, MEP, and handgrip strength significantly increased from D1 to D7 in both groups (p = 0.005, p = 0.007, and p = 0.028, respectively).

According to multiple linear regression, the need for IMV, female sex, and duration of IMV were independently and negatively associated with the SPPB score (R^2^ = 0.45) and handgrip strength (R^2^ = 0.59) at ICU discharge (Table 3). The SPPB score and handgrip strength were strongly correlated (r = 0.77; p ≤ 0.001).

**Table 3:** Multivariate analysis of variables associated with short physical performance battery and handgrip strength as the dependent variables

	SPPB			Handgrip strength		
	Beta	p value	R^2^	Beta	p value	R^2^
IMV need (yes/no)	-0.43	0.001	0.45	-0.47	≤ 0.001	0.59
Duration IMV (days)	-0.26	0.032		-0.29	0.006	
Female sex (yes/no)	-0.31	0.002		-0.41	≤ 0.001	
SOFA score on Day 5		0.38			0.06	
Corticosteroids use (days)		0.21			0.09	
Hemodialysis need (yes/no)		0.63			0.71	
Neuromuscular blockers use (yes/no)		0.93			0.48	

SPPB - short physical performance battery; IMV - invasive mechanical ventilation; SOFA - Sequential Organ Failure Assessment.

The median hospital stays were 17 [15 - 25] days and 42 [30 - 73] days for the IMV and Non-IMV Groups, respectively (p < 0.001).

## DISCUSSION

The main findings of this study were that physical function disability was highly prevalent in patients who recovered from critical COVID-19 pneumonia and it persisted for more than 7 days even in patients who did not receive IMV. Both groups improved slightly at D7, but the Non-IMV Group had moderate functional limitations, while the IMV Group still exhibited severe functional limitations based on the SPPB scores. The very poor physical function of both groups was also confirmed by their handgrip strength, which was extremely reduced, especially in the IMV Group. In agreement with these results, the prevalence of ICU-acquired weakness was 13% and 72% at D7 in the Non-IMV and IMV Groups, respectively.

In accordance with our results, the studies by Belli et al.^(2)^ and Paneroni et al.^(3)^ also revealed that patients who recovered from severe COVID-19 pneumonia had impaired physical function at ICU discharge, with a slight improvement at discharge. However, their study predated the release of the COVID-19 vaccine, which may explain why their patients were older and why fewer patients under IMV survived and couldn't be studied.

Although physical impairment in these patients might be multifactorial, SARS-CoV-2 infection can elicit distinctive inflammatory responses within skeletal muscles, potentially contributing to the observed muscle dysfunction.^(16)^ This dysfunction appears to be more severe and disproportionate than other recognized contributing factors, such as hypoxia.^(17,18)^ Skeletal muscle cells express angiotensin-converting enzyme 2 (ACE2), which binds to SARS-CoV-2 and likely makes skeletal muscles vulnerable to direct virus invasion, leading to muscle damage and reduced limb muscle mass.^(18)^ This might explain why our patients who were not invasively ventilated and had a mean age of 61 years still presented considerable physical functional disorders.

We also found that female sex, along with the need for and time spent under IMV, was independently associated with worse physical function performance. A greater prevalence of frailty in females than in males has been reported in the ICU,^(19)^ and female sex has been found to be significantly associated with ICU-acquired weakness.^(20,21)^ Possible explanations for this greater vulnerability are that women have less muscle mass but also that women have greater expression of ACE2 in skeletal muscle.^(17)^ Notably after accounting for the duration of IMV, the use of paralytic agents did not seem to contribute to further muscle disability (Table 3).

Interestingly, the MIP was better preserved than the MEP and handgrip strength in both groups, suggesting that the diaphragm might be more resistant to muscle inflammation damage. Sarcopenia is less common in muscles with a greater proportion of fatigue-resistant fibers.^(22)^ Given that the diaphragm primarily consists of fatigue-resistant fibers (55% type 1, 25% type 2A), discrepancies in muscle fiber-type composition may also contribute to this difference.^(23)^

It is important to acknowledge that due to the study design, we cannot assert that our patients with COVID-19 exhibited worse physical function than those with critical illnesses from other causes. A study by Hodgson et al. revealed no significant differences in the incidence or severity of new disability at 6 months after ICU admission between patients requiring IMV for acute respiratory distress syndrome caused by COVID-19 and those caused by reasons other than COVID-19.^(8)^ In another two studies, there were no differences in predicted distances during the six-minute walking distance test^(24)^ or in self-reported physical symptoms^(25)^ between COVID-19 ICU survivors and those unrelated to COVID-19.

There are several limitations to this study. First, critical information such as preadmission physical function and vaccination status was not documented. Second, the sample size was limited, and there were some patients that were lost to follow-up (17%). Third, we could not definitively exclude other potential factors contributing to disability following critical illness in the ICU, including preexisting disability, hyperglycemia, acute illness severity, and sepsis.^(20,26)^ Consequently, factors beyond SARS-CoV-2 infection may have influenced the magnitude and marginal recovery of physical function observed in our patients. However, our study strength lies in re-evaluating patients post-ICU discharge within the same timeframe, differing from prior research. This timing is crucial due to pandemic-related factors such as health insurance and COVID-19 concerns potentially influencing discharge decisions, introducing bias in post severe COVID-19 functional recovery assessments.

## CONCLUSION

Patients who recovered from critical COVID-19 and who received invasive mechanical ventilation presented greater disabilities than those who were not invasively ventilated. The recovery of physical functional disability during the 7-day follow-up in the ward was marginal, and notably, it did not significantly differ between patients requiring invasive mechanical ventilation and those who did not. This outcome may underscore the severity of functional impairment induced by SARS-CoV-2 infection.

The observed respiratory and functional impairments in hospitalized patients after critical COVID-19 indicate that a comprehensive assessment of functional performance of patients in the intensive care unit until discharge is imperative to guide rehabilitation.
