# Supplementary material for: A comprehensive physical functional assessment of survivors of critical care unit stay due to COVID-19
Source: Crit Care Sci. 2024 Apr 16;36:e20240284en. doi: 10.62675/2965-2774.20240284-en (PMC11098067; doi:10.62675/2965-2774.20240284-en)
Supplement: Translation

# Translation: Avaliação físico-funcional abrangente de sobreviventes de internação em unidade de terapia intensiva devido à COVID-19

**DOI:** 10.62675/2965-2774.20240284-pt

**Authors:** Marcia Souza Volpe, Ana Carolina Cardoso dos Santos, Sílvia Gaspar, Jade Lara de Melo, Gabriela Harada, Patrícia Rocha Alves Ferreira, Karina Ramiceli Soares da Silva, Natália Tiemi Simokomaki Souza, Carlos Toufen, Luciana Dias Chiavegato, Marcelo Britto Passos Amato, Maria Ignez Zanetti Feltrim, Carlos Roberto Ribeiro de Carvalho

**Affiliations:** 1 Santos SP Brasil Departamento de Ciências do Movimento Humano, Universidade Federal de São Paulo - Santos (SP), Brasil.; 2 São Paulo SP Brasil Divisão de Pneumologia, Instituto do Coração, Hospital das Clínicas, Faculdade de Medicina, Universidade de São Paulo - São Paulo (SP), Brasil.; 3 São Paulo SP Brasil Serviço de Fisioterapia, Instituto do Coração, Hospital das Clínicas, Faculdade de Medicina, Universidade de São Paulo - São Paulo (SP), Brasil.; 4 São Paulo SP Brasil Disciplina de Pneumologia, Universidade Federal de São Paulo - São Paulo (SP), Brasil.

**Keywords:** COVID-19, Infecções por coronavírus, Cuidados críticos, Recuperação de função fisiológica, Testes de função respiratória

## Abstract

**Objetivo:**

Examinar a função física e a força muscular respiratória de pacientes que se recuperaram da COVID-19 grave após a alta da unidade de terapia intensiva para a enfermaria nos Dias 1 e 7 e investigar as variáveis associadas ao comprometimento funcional.

**Métodos:**

Trata-se de estudo de coorte prospectivo de pacientes adultos com COVID-19 que necessitaram de ventilação mecânica invasiva, ventilação mecânica não invasiva ou cânula nasal de alto fluxo e tiveram alta da unidade de terapia intensiva para a enfermaria. Os participantes foram submetidos aos testes *Medical Research Council sum-score*, força de preensão manual, pressão inspiratória máxima, pressão expiratória máxima e *short physical performance battery*. Os participantes foram agrupados em dois grupos conforme a necessidade de ventilação mecânica invasiva: o Grupo Ventilação Mecânica Invasiva (Grupo VMI) e o Grupo Não Ventilação Mecânica Invasiva (Grupo Não VMI).

**Resultados:**

Os pacientes do Grupo VMI (n = 31) eram mais jovens e tinham pontuações do *Sequential Organ Failure Assessment* mais altas do que os do Grupo VMI (n = 33). As pontuações do *short physical performance battery* (intervalo de zero a 12) nos Dias 1 e 7 foram 6,1 ± 4,3 e 7,3 ± 3,8, respectivamente para o Grupo Não VMI, e 1,3 ± 2,5 e 2,6 ± 3,7, respectivamente para o Grupo VMI. A prevalência de fraqueza adquirida na unidade de terapia intensiva no Dia 7 foi de 13% para o Grupo Não VMI e de 72% para o Grupo VMI. A pressão inspiratória máxima, a pressão expiratória máxima e a força de preensão manual aumentaram no Dia 7 em ambos os grupos, porém a pressão expiratória máxima e a força de preensão manual ainda eram fracas. Apenas a pressão inspiratória máxima foi recuperada (ou seja, > 80% do valor previsto) no Grupo Não VMI. As variáveis sexo feminino, e necessidade e duração da ventilação mecânica invasiva foram associadas de forma independente e negativa à pontuação do *short physical performance battery* e à força de preensão manual.

**Conclusão:**

Os pacientes que se recuperaram da COVID-19 grave e receberam ventilação mecânica invasiva apresentaram maior incapacidade do que aqueles que não foram ventilados invasivamente. No entanto, os dois grupos de pacientes apresentaram melhora funcional marginal durante a fase inicial de recuperação, independentemente da necessidade de ventilação mecânica invasiva. Esse resultado pode evidenciar a gravidade da incapacidade causada pelo SARS-CoV-2.

## INTRODUÇÃO

A doença causada pelo coronavírus 2019 (COVID-19) com insuficiência respiratória aguda tem sido associada a internações prolongadas em unidades de terapia intensiva (UTI) e comprometimento funcional prolongado e persistente.^(1)^ No entanto, o comprometimento funcional de pacientes que sobrevivem a formas graves e críticas da COVID-19 é ainda pouco relatado, e a maioria dos dados provém da primeira onda da pandemia e inclui pacientes mais velhos.^(2–5)^ Além disso, grande parte dos estudos era retrospectiva, e há estudos limitados sobre a força muscular respiratória nessa população.^(6,7)^

Embora os casos críticos e moderados da COVID-19 tenham praticamente cessado, tem-se sugerido que o comprometimento funcional entre os sobreviventes da COVID-19 nas UTIs pode não diferir substancialmente daquele entre os sobreviventes que se recuperam de doenças críticas causadas por outros fatores.^(8)^ Consequentemente, a compreensão da função respiratória e física de pacientes nas fases iniciais de recuperação da COVID-19 grave poderia orientar o desenvolvimento de intervenções e terapias para apoiar a reabilitação não apenas de pacientes que se recuperam da COVID-19, mas também daqueles que se recuperam de doenças críticas não relacionadas à COVID-19.

O objetivo principal deste estudo foi examinar a função física e a força muscular respiratória de pacientes que se recuperaram da COVID-19 grave após a alta da UTI para a enfermaria nos Dias 1 e 7. O objetivo secundário foi investigar as variáveis associadas ao comprometimento físico.

## MÉTODOS

Realizamos um estudo de coorte prospectivo em dois hospitais brasileiros, o Instituto do Coração do Hospital das Clínicas da Faculdade de Medicina da Universidade de São Paulo (USP) e o Hospital São Paulo da Universidade Federal de São Paulo (UNIFESP), Brasil. Este estudo foi aprovado pelos comitês de ética de ambos os hospitais (Hospital das Clínicas da Faculdade de Medicina da USP: n° 4.711.382; e UNIFESP: n° 4.870.812). Obteve-se o consentimento informado de todos os pacientes.

Devido à natureza exploratória do estudo, não foi calculado o tamanho amostral, e utilizou-se uma amostra de conveniência. Os critérios de inclusão foram pacientes internados na UTI devido à infecção por COVID-19 confirmada em laboratório, idade ≥ 18 anos, necessidade de ventilação mecânica invasiva (VMI), ventilação não invasiva (VNI) ou cânula nasal de alto fluxo e alta da UTI para a enfermaria. Os critérios de exclusão foram histórico de amputação da mão do membro superior dominante, distúrbios cognitivos anteriores e permanentes ou deficiências neuromusculoesqueléticas, incapacidade de consentir em participar do estudo e/ou incapacidade de concluir as avaliações físicas propostas. A inclusão ocorreu entre julho de 2021 e fevereiro de 2022.

Os participantes do estudo foram submetidos às seguintes avaliações no primeiro dia (D1) na enfermaria após a alta da UTI e no sétimo dia (D7) - ou antes, se o paciente recebesse alta hospitalar: s*hort physical performance battery* (SPPB), *Medical Research Council sum-score* (MRC-SS), escala de mobilidade da UTI, força de preensão manual, pressão inspiratória máxima (PImáx) e pressão expiratória máxima (PEmáx).

O SPPB combina três testes funcionais: equilíbrio em pé, velocidade de marcha de 3m e sentar e levantar com cinco repetições. As pontuações do SPPB de zero a 3 indicam comprometimento grave; de 4 a 6 indicam função baixa; de 7 a 9 indicam função intermediária; e de 10 a 12 indicam função normal.^(9)^ O MRC-SS varia de zero (paralisia total) a 60 (força normal) e foi usado para avaliar a força muscular geral de acordo com um protocolo padronizado.^(10)^ A escala de mobilidade na UTI é uma escala de 11 pontos usada para medir o nível mais alto de mobilidade funcional dos pacientes, em que zero significa nenhuma mobilidade e dez significa caminhar independentemente sem auxílio para a marcha.^(11)^ A força de preensão manual da mão dominante foi avaliada de acordo com um protocolo padronizado^(12)^ e foi relatada como percentual dos valores de referência.^(13)^ Os valores de PImáx e PEmáx foram avaliados segundo as diretrizes da *American Thoracic Society* (ATS)^(14)^ e são relatados como percentuais dos valores previstos.^(15)^

Os participantes foram agrupados quanto à necessidade de VMI em: Grupo VMI e Grupo Não VMI. As variáveis categóricas são relatadas como contagens e percentuais. As variáveis contínuas são relatadas como média e desvio-padrão ou medianas e intervalos interquartis, conforme a distribuição. As variáveis categóricas foram comparadas usando o teste do qui-quadrado ou o teste exato de Fisher, conforme o caso. As variáveis contínuas foram comparadas por meio de testes *t*, testes de Wilcoxon-Mann-Wann ou análise de variância para medidas repetidas, conforme recomendação. O modelo de análise de variância foi construído com um fator intragrupo (tempo: D1 *versus* D7) e um fator intergrupos (Grupos VMI *versus* Não VMI). As variáveis significativas (p ≤ 0,05) foram incluídas nos modelos de análise de regressão múltipla para investigar os preditores da função física com base no SPPB e na força de preensão manual. Os dados faltantes não foram imputados. Considerou-se um valor de p bilateral < 0,05 para indicar significância estatística.

## RESULTADOS

Foram incluídos 64 pacientes no estudo, 33 dos quais necessitavam de VMI. No D7, 11 pacientes (7 do Grupo Não VMI e 4 do Grupo VMI) não foram avaliados devido à alta hospitalar não programada. A tabela 1 apresenta os dados demográficos e as características clínicas dos participantes de ambos os grupos. Os pacientes do Grupo VMI eram mais jovens, apresentavam pontuações SOFA mais altas e tinham menos hipertensão do que os pacientes que não precisavam de VMI.

**Tabela 1:** Dados demográficos e características clínicas dos participantes dos Grupos Ventilação Mecânica Invasiva e Não Ventilação Mecânica Invasiva

		Não VMI (n = 31)	VMI (n = 33)	Valor de p
Idade (anos)		61,4 ± 14,3	51,3 ± 15,5	0,009*
Peso (kg)		80,3 ± 18,3	83,1 ± 22,4	
IMC (kg/m^2^)		28,8 ± 5,0	28,9 ± 6,7	
Masculino (sexo)		19 (61,3)	21 (63,6)	
Comorbidades				
	Hipertensão	16 (51,6)	7 (21,2)	0,018†
	Obesidade	7 (22,6)	12 (36,4)	
	Doença cardiovascular	8 (25,8)	4 (12,1)	
	Dislipidemia	10 (32,3)	8 (24,2)	
	Histórico de tabagismo	10 (32,3)	7 (21,2)	
	Doença renal	5 (16,1)	10 (30,3)	
	DPOC	3 (9,7)	2 (6,1)	
Número de comorbidades				
	0	2 (6,5)	6 (18,2)	
	1	12 (38,7)	13 (39,4)	
	2	6 (19,4)	8 (24,2)	
	3	8 (25,8)	3 (9,1)	
	≥ 4	3 (9,7)	3 (9,1)	
	Necessidade de hemodiálise	2 (6,5)	12 (36,4)	0,006†
	SOFA, dia 1 na UTI	2 [2 - 4]	7 [3 - 10,5]	< 0,001‡
	SOFA, dia 5 na UTI	2 [2 - 5]	7 [3 - 11]	< 0,001‡
	Uso de corticosteroides§ (dias)	13 [9 - 18]	25 [16 - 30,0]	< 0,001‡
	Internação na UTI (dias)	11 [8 - 16]	29 [16 -47,5]	< 0,001‡

VMI - ventilação mecânica invasiva; IMC - índice de massa corporal; DPOC - doença pulmonar obstrutiva crônica; SOFA - *Sequential Organ Failure Assessment*; UTI - unidade de terapia intensiva.

* Teste *t*;

† teste exato de Fisher ou teste do qui-quadrado;

‡ teste U de Mann-Whitney;

§ a avaliação do uso de corticosteroides foi limitada a 30 dias de internação hospitalar. Resultados expressos como média ± desvio-padrão, n (%) ou mediana [intervalo interquartil].

No Grupo Não VMI, a maioria (54,8%) dos pacientes usou cateter nasal de alto fluxo (CNAF), seguida por CNAF intercalada com VNI (35,5%) e uma minoria (9,7%) usou apenas VNI. A duração média da terapia com CNAF foi de 6 [4 - 8] dias, enquanto a VNI durou 3 [1 - 8] dias. No Grupo VMI, 72,7% dos pacientes exigiram uma média de 2,0 (1,0) sessões de posicionamento em decúbito ventral. A duração média do uso de bloqueadores neuromusculares durante a VMI controlada foi de 3,0 [1 - 7] dias, e foi de 0,5 [0 - 3] dias durante a VMI assistida. A traqueostomia foi realizada em 42,4% dos pacientes, e a duração da VMI foi de 15 [9 - 38] dias.

A tabela 2 apresenta os resultados das avaliações físicas dos pacientes de ambos os grupos nos dias 1 e 7 na enfermaria após a alta da UTI. Em ambos os grupos, houve leve melhora na pontuação SPPB ao longo do tempo (p ≤ 0,001, fator tempo), mas a pontuação SPPB ainda era ruim, especialmente no Grupo VMI (p ≤ 0,001, fator grupo). A figura 1 apresenta um diagrama com as subpontuações dos três testes funcionais - equilíbrio em pé, velocidade da marcha e sentar e levantar, que compõem a pontuação total do SPPB. Claramente, as pontuações mais baixas para ambos os grupos foram do teste de sentar e levantar.

**Tabela 2:** Resultados das avaliações de função física nos dias 1 e 7 no Grupo Ventilação Mecânica Invasiva e no Grupo Não Ventilação Mecânica Invasiva

	Não VMI (n = 24)		VMI (n = 29)		Valor de p*	Valor de p†
	Dia 1	Dia 7	Dia 1	Dia 7		
SPPB (pontos)	6,1 ± 4,3	7,3 ± 3,8	1,3 ± 2,5	2,6 ± 3,7	≤ 0,001	≤ 0,001
MRC-SS (pontos)	54,7 ± 7,3	55,8 ± 6,4	42,6 ± 11,0	43,7 ± 10,6	≤ 0,001	0,209
Mobilidade na UTI (pontos)	8,0 ± 2,3	9,2 ± 1,9	4,6 ± 2,3	6,1 ± 2,9	≤ 0,001	≤ 0,001
FPM, % do previsto	53,0 ± 20,5	54,0 ± 21,2	24,1 ± 17,1	30,0 ± 16,8	≤ 0,001	0,005
PImáx, % do previsto	85,9 ± 39,5	99,1 ± 37,1	55,3 ± 31,1	64,5 ± 33,4	0,002	0,007
PEmáx, % do previsto	56,4 ± 28,7	66,3 ± 24,6	36,7 ± 20,2	42,9 ± 20,2	0,001	0,028

VMI - ventilação mecânica invasiva; SPPB – *short physical performance battery*; MRC-SS - *Medical Research Council sum-score*; UTI - unidade de terapia intensiva; FPM - força de preensão manual; PImáx - pressão inspiratória máxima; PEmáx - pressão expiratória máxima.

* Análise de variância para medidas repetidas, fator intergrupo (Grupos Ventilação Mecânica Invasiva *versus* Não Ventilação Mecânica Invasiva);

† análise de variância para medidas repetidas, fator intragrupo (tempo, dia 1 *versus* dia 7). Resultados expressos como média ± desvio-padrão.

**Figura 1:** Diagrama do *short physical performance battery* mostrando as subpontuações de três testes - equilíbrio em pé, velocidade da marcha e sentar e levantar - de ambos os grupos nos Dias 1 e 7.

A pontuação de cada teste varia de zero a quatro, e a pontuação total da *short physical performance battery* varia de zero a 12. As pontuações entre zero e 3 denotam comprometimento grave da função física, as pontuações entre 4 e 6 indicam baixa função, as pontuações entre 7 e 9 indicam função intermediária e as pontuações entre 10 e 12 indicam função normal. As pontuações do Grupo Ventilação Mecânica Invasiva são apresentadas em cinza, e as pontuações do Grupo Não Ventilação Mecânica Invasiva são apresentadas em azul. As pontuações no Dia 1 são mostradas como linhas contínuas, e as pontuações no Dia 7 são mostradas como linhas tracejadas. Os dados são apresentados como média ± desvio padrão.

VMI - ventilação mecânica invasiva; SPPB - *short physical performance battery*; D1 - Dia 1; D7 - Dia 7.

Nenhum dos grupos apresentou aumento significativo nas pontuações MRC-SS ao longo do tempo (p = 0,209). A incidência de fraqueza adquirida na UTI (MRC-SS < 48) foi de 13% no Dia 7 para o Grupo Não VMI, enquanto no Grupo VMI foi de 72%.

A pontuação da escala de mobilidade na UTI aumentou em ambos os grupos (p ≤ 0,001, fator tempo). No Grupo Não VMI, a pontuação média variou de "caminhar com a ajuda de uma pessoa" a "caminhar independentemente com auxílio para a marcha", e no Grupo VMI, a pontuação média variou de "transferir-se da cama para a cadeira" a "marchar localmente ao lado da cama" (Tabela 2).

É interessante notar que a PImáx foi menos afetada, enquanto a PEmáx e a força de preensão manual ficaram bem abaixo de 80% dos valores preditos, especialmente no grupo VMI (Tabela 2). A PImáx, a PEmáx e a força de preensão manual aumentaram significativamente do D1 ao D7 em ambos os grupos (p = 0,005, p = 0,007 e p = 0,028, respectivamente).

De acordo com a regressão linear múltipla, a necessidade de VMI, o sexo feminino e a duração da VMI foram independentemente e negativamente associados à pontuação do SPPB (R^2^ = 0,45) e à força de preensão manual (R^2^ = 0,59) na alta da UTI (Tabela 3). A pontuação do SPPB e a força de preensão manual apresentaram forte correlação (r = 0,77; p ≤ 0,001).

**Tabela 3:** Análise multivariada das variáveis associadas ao *short physical performance battery* e à força de preensão manual como variáveis dependentes

	SPPB			Força de preensão manual		
	Beta	Valor de p	R^2^	Beta	Valor de p	R^2^
Necessidade de VMI (sim/não)	-0,43	0,001	0,45	-0,47	≤ 0,001	0,59
Duração da VMI (dias)	-0,26	0,032		-0,29	0,006	
Sexo feminino (sim/não)	-0,31	0,002		-0,41	≤ 0,001	
SOFA no dia 5		0,38			0,06	
Uso de corticosteroides (dias)		0,21			0,09	
Necessidade de hemodiálise (sim/não)		0,63			0,71	
Uso de bloqueadores neuromusculares (sim/não)		0,93			0,48	

SPPB - *short physical performance battery*; VMI - ventilação mecânica invasiva; SOFA - *Sequential Organ Failure Assessment*.

A média de internação hospitalar foi de 17 [15 - 25] dias e 42 [30 - 73] dias para os Grupos VMI e Não VMI, respectivamente (p < 0,001).

## DISCUSSÃO

Os principais achados deste estudo foram que a incapacidade física era altamente prevalente em pacientes que se recuperaram de pneumonia crítica por COVID-19 e que persistiu por mais de 7 dias, mesmo em pacientes que não receberam VMI. Ambos os grupos melhoraram ligeiramente no D7, mas o Grupo Não VMI apresentou limitações funcionais moderadas, enquanto o Grupo VMI ainda apresentava limitações funcionais graves com base nas pontuações do SPPB. A função física muito ruim de ambos os grupos também foi confirmada por sua força de preensão manual, que era extremamente reduzida, especialmente no Grupo VMI. A prevalência de fraqueza adquirida na UTI de 13% e 72% no dia 7 nos Grupos Nao VMI e VMI, respectivamente, também reforça esses achados.

Assim como nos nossos resultados, os estudos de Belli et al.^(2)^ e Paneroni et al.^(3)^ também revelaram que os pacientes que se recuperaram de pneumonia grave por COVID-19 apresentaram comprometimento da função física na alta da UTI, com leve melhora na alta hospitalar. No entanto, seus estudos foram anteriores à disponibilização da vacina contra a COVID-19, o que pode explicar por que seus pacientes eram mais velhos e por que menos pacientes sob VMI sobreviveram e não puderam ser estudados.

Embora o comprometimento físico desses pacientes possa ser multifatorial, a infecção pelo coronavírus da síndrome respiratória aguda grave 2 (SARS-CoV-2) pode provocar respostas inflamatórias distintas nos músculos esqueléticos, contribuindo potencialmente para a disfunção muscular observada.^(16)^ Essa disfunção parece ser mais grave e desproporcional do que outros fatores contribuintes reconhecidos, como a hipóxia.^(17,18)^ As células musculares esqueléticas expressam a enzima conversora de angiotensina 2 (ACE2), que se liga ao SARS-CoV-2 e, provavelmente, torna os músculos esqueléticos vulneráveis à invasão direta do vírus, levando a danos musculares e à redução da massa muscular dos membros.^(18)^ Isso pode explicar por que nossos pacientes que não foram ventilados de forma invasiva e tinham idade média de 61 anos ainda apresentavam distúrbios físicos funcionais consideráveis.

Também encontramos que o sexo feminino, além da necessidade e do tempo de permanência em VMI, foi independentemente associado a um desempenho pior da função física. Há relatos na literatura de uma maior prevalência de fragilidade em mulheres do que em homens na UTI,^(19)^ e o sexo feminino mostrou-se significativamente associado à fraqueza adquirida na UTI.^(20,21)^ Uma possível explicação para essa maior vulnerabilidade é que as mulheres têm menos massa muscular, além de terem maior expressão de ACE2 no músculo esquelético.^(17)^ Notavelmente, depois de levar em conta a duração da VMI, o uso de agentes paralisantes não pareceu contribuir para maior incapacidade muscular (Tabela 3).

É interessante notar que a PImáx foi mais bem preservada do que a PEmáx e a força de preensão manual em ambos os grupos, sugerindo que o diafragma pode ser mais resistente a danos causados pela inflamação muscular. A sarcopenia é menos comum em músculos com maior proporção de fibras resistentes à fadiga.^(22)^ Considerando que o diafragma consiste principalmente de fibras resistentes à fadiga (55% tipo 1, 25% tipo 2A), discrepâncias na composição do tipo de fibra muscular também podem contribuir para essa diferença.^(23)^

É importante reconhecer que, devido ao desenho do estudo, não podemos afirmar que nossos pacientes com COVID-19 apresentaram pior função física do que aqueles com doenças críticas de outras causas. Um estudo realizado por Hodgson et al. não revelou diferenças significativas na incidência ou gravidade de novas incapacidades 6 meses após a admissão na UTI entre pacientes que necessitaram de VMI para síndrome do desconforto respiratório agudo causada pela COVID-19 e aqueles causados por outras razões que não a COVID-19.^(8)^ Em outros dois estudos, não houve diferenças nas distâncias previstas durante o teste de caminhada de 6 minutos^(24)^ ou nos sintomas físicos autorrelatados^(25)^ entre os sobreviventes da COVID-19 na UTI e aqueles não relacionados à COVID-19.

Este estudo apresenta várias limitações. Em primeiro lugar, não foram documentadas informações essenciais, como a função física antes da internação e a condição vacinal. Em segundo lugar, o tamanho da amostra foi limitado e alguns pacientes foram perdidos no seguimento (17%). Terceiro, não pudemos excluir definitivamente outros possíveis fatores que contribuem para a incapacidade após uma doença crítica na UTI, incluindo incapacidade preexistente, hiperglicemia, gravidade da doença aguda e sepse.^(20,26)^ Consequentemente, fatores além da infecção por SARS-CoV-2 podem ter influenciado a magnitude e a recuperação marginal da função física observada em nossos pacientes. No entanto, um ponto forte do nosso estudo está na reavaliação dos pacientes após a alta da UTI dentro do mesmo período de tempo, diferindo de pesquisas anteriores. Esse momento é crucial devido a fatores relacionados à pandemia, como seguro de saúde e preocupações com a COVID-19, que podem ter influenciado as decisões de alta, introduzindo viés nas avaliações de funcionalidade pós-COVID-19 grave.

## CONCLUSÃO

Os pacientes que se recuperaram da COVID-19 crítica e que receberam ventilação mecânica invasiva apresentaram maiores incapacidades do que aqueles que não foram ventilados invasivamente. A recuperação da incapacidade física funcional durante o acompanhamento de 7 dias na enfermaria foi marginal e, notavelmente, não diferiu significativamente entre os pacientes que necessitaram de ventilação mecânica invasiva e aqueles que não necessitaram de ventilação mecânica invasiva. Esse resultado pode ressaltar a gravidade da incapacidade funcional induzida pela infecção por SARS-CoV-2.

As deficiências respiratórias e funcionais observadas em pacientes hospitalizados após a COVID-19 crítica indicam que uma avaliação abrangente da funcionalidade dos pacientes na unidade de terapia intensiva até a alta é imperativa para orientar a reabilitação.
